# Pupil Diameter Tracks Lapses of Attention

**DOI:** 10.1371/journal.pone.0165274

**Published:** 2016-10-21

**Authors:** Ruud L. van den Brink, Peter R. Murphy, Sander Nieuwenhuis

**Affiliations:** 1 Institute of Psychology, Leiden University, Leiden, the Netherlands; 2 Leiden Institute for Brain and Cognition (LIBC), Leiden, the Netherlands; 3 Department of Neurophysiology and Pathophysiology, University Medical Center Hamburg-Eppendorf, Hamburg, Germany; University of Hyderabad, INDIA

## Abstract

Our ability to sustain attention for prolonged periods of time is limited. Studies on the relationship between lapses of attention and psychophysiological markers of attentional state, such as pupil diameter, have yielded contradicting results. Here, we investigated the relationship between tonic fluctuations in pupil diameter and performance on a demanding sustained attention task. We found robust linear relationships between baseline pupil diameter and several measures of task performance, suggesting that attentional lapses tended to occur when pupil diameter was small. However, these observations were primarily driven by the joint effects of time-on-task on baseline pupil diameter and task performance. The linear relationships disappeared when we statistically controlled for time-on-task effects and were replaced by consistent inverted U-shaped relationships between baseline pupil diameter and each of the task performance measures, such that most false alarms and the longest and most variable response times occurred when pupil diameter was both relatively small and large. Finally, we observed strong linear relationships between the temporal derivative of pupil diameter and task performance measures, which were largely independent of time-on-task. Our results help to reconcile contradicting findings in the literature on pupil-linked changes in attentional state, and are consistent with the adaptive gain theory of locus coeruleus-norepinephrine function. Moreover, they suggest that the derivative of baseline pupil diameter is a potentially useful psychophysiological marker that could be used in the on-line prediction and prevention of attentional lapses.

## Introduction

The ability to sustain attention for prolonged periods of time is essential for normal functioning in everyday life. Lapses of attention can have dramatic consequences, such as when a car driver is absent-minded and brakes too late in response to an unexpected traffic backup, or when an air traffic controller fails to spot that two aircraft are about to cross paths. Physiological markers that indicate when such lapses of attention are more likely to occur could yield insight into the cognitive mechanisms that underlie attentional lapses, as well as provide preventative measures. A potentially useful physiological marker for detecting lapses of attention might be pupil diameter. The diameter of the pupil has long been known as a marker of cognitive load and attentional performance [1,2]. More recently, some researchers have considered endogenous (‘baseline’) variability in pupil diameter as an indicator of fluctuations in attentional control state (e.g., [3–5]).

Despite the potential utility of pupil diameter as a marker of attentional engagement, the available studies in which the relationship between baseline pupil diameter and sustained attentional performance has been investigated display a remarkable lack of consistency. Some researchers have reported that moments of poor task performance or off-task thought are associated with larger baseline diameter [6–9]. Others have reported that poor task performance is associated with *smaller* baseline diameter [10–14], or is preceded by a progressive decline in pupil diameter [10,15]. Finally, some studies have found that poor task performance can be accompanied by both relatively small and relatively large baseline diameter [7,9,15,16]. Several theoretical and methodological factors may be responsible for this discrepancy. For instance, the studies reviewed here differed considerably with regard to the measures they used to assess attentional performance: response time (RT; [7,8]); a proportion of slowest responses [9,12]; variability in RTs [15]; perceptual sensitivity [11,17,18]; and self-reported off-task thought [6,10,13].

Another factor that may contribute to the lack of consistency in this literature concerns time-on-task effects. Prolonged task performance often results in decrements in attentional performance due to reduced vigilance [19,20], and concurrent changes in pupil diameter [9,11,14]. For instance, Hopstaken and colleagues found a progressive decrease in both baseline diameter and perceptual sensitivity with prolonged performance of an N-back task [11]. A similar decline in both pupil diameter and performance was reported by Van Orden and colleagues, using a sustained attention task [14]. However, in other studies time-on-task has been reported to lead to contrasting effects on pupil diameter and task performance. For instance, Murphy and colleagues found a progressive *increase* over time in baseline diameter during an oddball task and a trend towards poorer performance over time [15]. Beatty reported a decrement over time in perceptual sensitivity during an oddball task, but no change in baseline diameter [17]. These time-on-task effects are often not taken into account when assessing the relationship between pupil diameter and performance (but see Kristjansson et al. [12] and Mathôt et al. [21] for notable exceptions). Thus, depending on the behavioral task and context, shared effects of time-on-task could in principle impose a relationship between diameter and task performance, or obscure a more nuanced relationship.

An example of such a nuanced relationship is the Yerkes-Dodson law, the phenomenon that performance often varies as an inverted-U function of arousal, such that both under- and over-arousal are associated with poor performance [22]. Aston-Jones and Cohen [3], in their adaptive gain theory, proposed that this relationship reflects the effects of neuromodulation originating from the locus coeruleus-norepinephrine (LC-NE) system. The LC is a small nucleus in the pontine tegmentum that collateralizes broadly and supplies NE to almost the entire brain [3,23]. Over longer time periods, the level of baseline activity of LC neurons fluctuates with task performance. Intermediate levels of baseline LC activity are associated with (near-)optimal performance, whereas shifts toward either end of the baseline activity continuum are associated with declining performance [3,24–26]. Notably, activity in the LC has been reported to correlate with the size of the pupil [3,27–29]. Thus, taken together, this framework predicts that both periods of small baseline diameter and periods of large baseline diameter should be associated with impaired attentional performance. Unfortunately, most studies so far have been confined to categorical comparisons of pupil diameter between on-task and off-task thought, or fast and slow response times, without taking nonlinear relationships into consideration.

Thus, in the present study we carried out a detailed investigation of the interrelationships between performance on a sustained attention task, slow baseline fluctuations in the diameter of the pupil, and the effects of time-on-task on both these variables. In contrast to some previous studies, we assessed these relationships at a within-participant, moment-by-moment level, using multiple measures of attentional state. This approach requires large numbers of trials, which in many studies is made difficult by the fact that short intertrial intervals can lead to contamination of pre-trial baseline pupil measurements by task-related pupil dilations on the previous trial. Here, we overcame this challenge by using a fast-paced, isoluminant, gradual-onset continuous performance task [30] to minimize stimulus-evoked pupil dilations, and by regressing out the remaining task-related transient pupil dilations.

Our results show that attentional performance and baseline diameter progressively declined over time, resulting in strong linear relationships between these variables. However, when we controlled for time-on-task, the relationships between task performance and pupil diameter became U-shaped, consistent with the Yerkes-Dodson law and the adaptive gain theory of LC function [3]. Moreover, we explored the relationship between performance and changes in pupil size quantified as the temporal derivative of baseline diameter. This measure was inspired by prior work in rodents, showing that the derivative of pupil diameter tracks changes in cortical state and signal detection performance [16,31]. As opposed to baseline diameter, its derivative showed a linear relationship with behavioral performance that was robust to the effect of time-on-task.

## Materials and Methods

### Participants

A total of 30 right-handed individuals took part in the study. Two participants were excluded due to technical difficulties with the eye tracker, resulting in a final N of 28 (mean age: 20.9; SD 2.5; min/max 18–26; 6 male). Exclusion criteria included a history of psychiatric disorders or wearing glasses. All participants gave written informed consent prior to the experiment and were compensated with €7,50 or course credit. The study was approved by the Leiden University Institutional Review Board (IRB).

### Task

**Fig 1:**
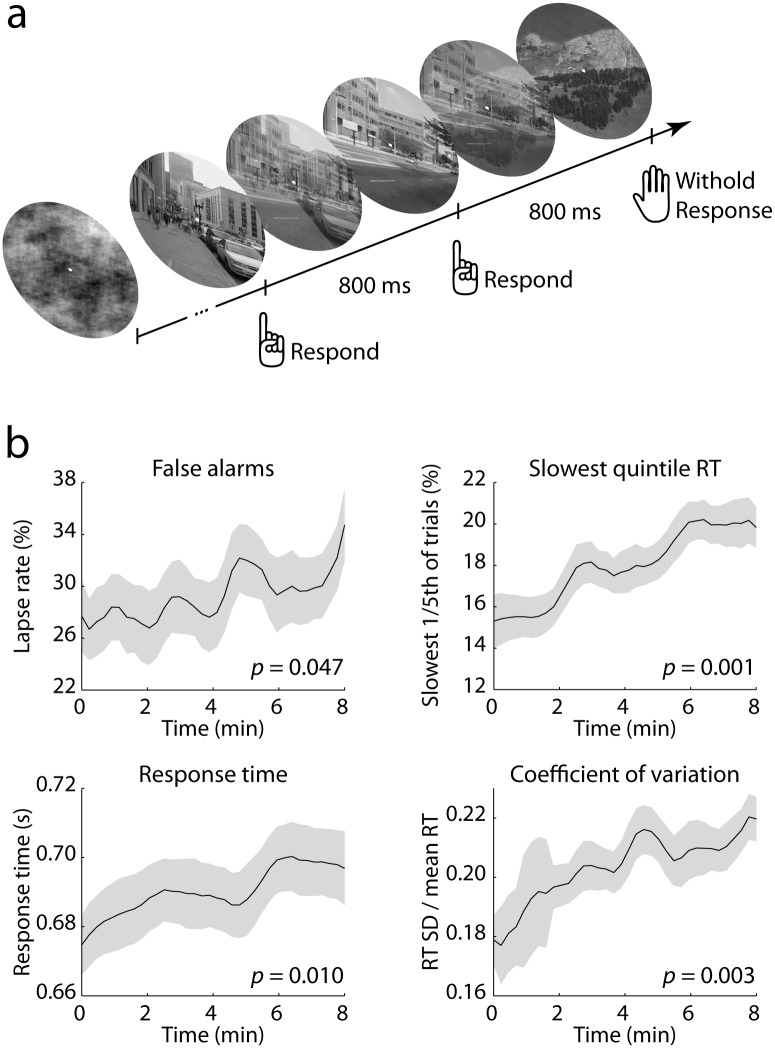
Task and behavioral results. a) Gradual continuous performance task. Each block started and ended with 7 scrambled images. The participant was asked to respond to city scenes but not to mountain scenes. Each image continuously morphed into the next, with an 800-ms interval between 100% coherence levels. b) Behavioral results. Data are smoothed for display only. All measures showed a significant linear increase with time-on-task, *p-*values are listed in the lower right corner of each panel. Error bars represent the SEM.

We used a modified version of the gradual continuous performance task (gradCPT) described by Esterman *et al*. [30]. Participants were asked to respond to images of cities by pressing the space bar and withhold a response when presented with an image of a mountain (Fig 1a). City trials were more frequent (90% of trials) than mountain trials (10%). The images subtended approximately 6 degrees of visual angle, were isoluminant, grayscale, and were presented on a black background. The images linearly and continuously morphed from one into the next, with an 800-ms interval between 100% coherence levels (stimulus onset asynchrony, SOA). This was done to provide a task context in which the participant had to continuously monitor the stimulus stream, and thus could not take ‘mini breaks’ in between trials. To allow the pupil to normalize, the first and last seven images in each block were scrambled. On these trials the participant did not respond and these trials were not included in any of the analyses.

Participants were first familiarized with the environment and task by passively viewing all images, without continuous transitions. Then, they practiced the task for 34 trials at ~45% of the normal speed, and then for another 75 trials at the regular speed. Each participant performed a total of 3 blocks of 600 trials (~8 minutes each) per block. Participants took a forced break of at least 5 minutes between blocks and were offered a small snack (chocolate chip cookie) during this interval. The total duration of the experiment was approximately 40 minutes.

### Measures of task performance

Hits (responses to cities) and correct rejections (withheld responses to mountains) were considered as correct trials. Misses constituted withheld responses to cities. Within the context of continuous performance tasks, lapses of attention are usually defined as false alarms [30,32]. In our study false alarms corresponded to responses to mountains. However, as noted in the introduction, a variety of other measures have been used to infer attentional state. Therefore, besides false alarm rate, we included three additional performance metrics: 1) the proportion of trials that fell within the slowest quintile of RTs within the block; 2) average RT; and 3) the RT coefficient of variation (RTCV)—that is, the standard deviation of RT divided by the block mean RT.

RTs were measured relative to the onset of each stimulus. For example, an RT of 640 ms (i.e., 80% of the SOA) indicated that the participant responded when the displayed image consisted of 80% trial *n*, and 20% trial *n*-1. An iterative algorithm assigned responses to trials in the case of multiple responses, or unusually fast responses (before 70% coherence of trial *n*) and unusually slow responses (after 40% coherence of trial *n*+1). First, the number of correct responses was optimized. Then, ambiguous responses were assigned to a neighboring trial if either of them had no response. If both had a response, it was assigned to the closest city (go) trial. Lastly, if a trial was assigned multiple responses, the fastest response was selected. This procedure was identical to the one described by Esterman *et al*. [30], and is unlikely to have substantially influenced the results, given that ambiguous responses were relatively rare (<4% of the trials).

### Pupillometry

Participants were seated in a dimly lit room with their head stabilized by a chin rest. During the task participants were asked to keep their eyes focused on a small white fixation dot in the center of the image. We measured the diameter of the right pupil at a sampling rate of 1 kHz with an EyeLink 1000 eye tracker. Prior to the start of each block the eye tracker was calibrated and validated with a 9-point fixation routine.

Moments when the eye tracker received no pupil signal (e.g., during blinks) were marked automatically during data acquisition by the manufacturer’s blink detection algorithm. Afterwards, an iterative algorithm detected additional moments of poor signal quality (e.g., due to partial occlusion of the pupil by the eyelashes). For 200 iterations over the entire signal time series for a given participant and block, any sample for which the difference in pupil diameter compared to the previous sample exceeded a threshold was marked as 0. The default threshold was set to 25 pixels, but the threshold was individually-tailored for participants for whom the algorithm failed to identify sharp spikes in the data or inappropriately marked clean sections of data. All marked data sections were then interpolated across using shape-preserving piecewise cubic interpolation. On average 6.8% (SD 4.6, min/max 0.2/18.7) of the data points were interpolated. After interpolation each pupil time series was low-pass filtered at 6 Hz to remove any residual high-frequency noise.

We were primarily interested in the relationship between tonic (endogenous) variations in pupil diameter and behavioral performance. Due to the short SOA of the gradCPT (800 ms), it is possible that stimulus-related pupil dilations precluded a reliable estimation of tonic pupil fluctuations. However, we first note that stimulus-related pupil responses accounted on average for only ~8% (SD 4%) of total pupil fluctuations, indicating that tonic fluctuations were a far more dominant source of variance in the observed pupil time series. Moreover, we reduced this already small contribution of trial-related pupil responses by employing linear regression to calculate residualized pupil time series for each participant and block that represented fluctuations in pupil diameter that were independent of the phasic pupil dilations evoked by task stimuli and their associated behavioral responses. The measured pupil time series were segmented around the onset of each stimulus, distinguishing between the four trial types (hits, misses, correct rejections, and false alarms), and around response onset. For each participant, we then computed average stimulus-locked and response-locked pupil waveforms, and extracted the peak amplitude in a 0 to 5 s post-event window, relative to a 200-ms pre-event baseline. This resulted in an estimate of the amplitude of phasic pupil dilations for each participant and type of event. Next, for each participant we created separate stick functions for each type of event in which the latency of the sticks corresponded to stimulus onsets and the participant’s RTs, and the amplitude corresponded to the estimated amplitude of the phasic pupil dilation for that participant and type of event. We then convolved the stick functions with the canonical pupillary response function (*h*) presented by Hoeks and Levelt [33]:

$$h=s\cdot (t^{n})\cdot e^{\left(\frac{-n\cdot t}{t_{\text{max}}}\right)}$$

where *t* is time, *n* is the number of layers (10.1), *t*_max_ corresponds to the latency of maximum dilatory response per participant and type of event, and *s* was a constant (2.7569·10^−29^) to scale the response function to unit height.

**Fig 2:**
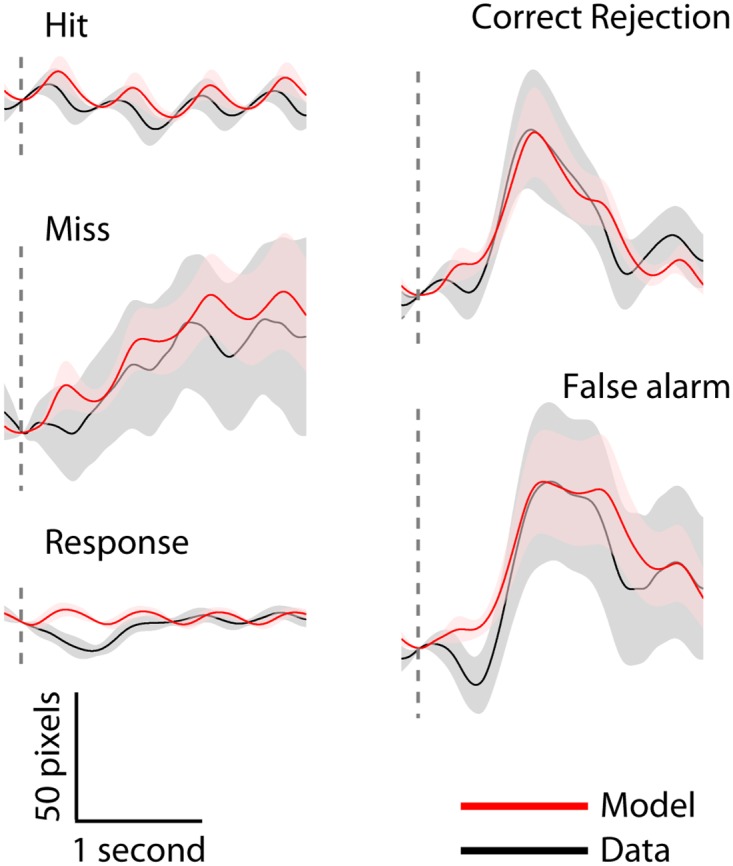
Phasic pupillary responses. Trial-averaged modeled (red) and empirical (black) stimulus-related pupil dilations. The vertical dashed line represents event (stimulus or response) onset. Error bars represent the SEM.

Finally, we used multiple linear regression to remove the stimulus- and response-related phasic dilations (Fig 2) from the unsegmented pupil time series. This procedure minimized the extent to which phasic pupil dilations convoluted the estimates of tonic variations in the diameter of the pupil. Note that this approach is highly similar to analysis of the first-stage general linear model of functional magnetic resonance imaging data, to correct for signal variance associated with trial-type-specific evoked responses (as implemented by e.g. [30]).

## Results

### Performance decrements with time-on-task

We first verified whether behavioral performance degraded over the course of a block, as is expected in demanding tasks like the gradCPT that require continually sustained attention [30,32]. To do so, we calculated temporally resolved metrics of trial-averaged behavior by applying a sliding window to the behavioral data of each block of each participant. The window had a width of 50 trials (40 seconds duration) and was slid across the data in steps of 15 trials. For each of these windows we calculated several measures of task performance: 1) the proportion of false alarms; 2) the proportion of trials that fell within the slowest quintile of RTs within the block; 3) average RT; and 4) the RTCV (see Materials & Methods). For each of these measures this approach resulted in a continuous time series for each block. We then Z-scored the time series and fitted a straight line to them. The slopes of the fitted lines indicated whether the time series were on average increasing or decreasing (or not changing) over time. We averaged the slopes across blocks for each participant and tested if the distribution of slopes was larger than 0 using a one-tailed *t*-test. As expected, we found significant performance decrements for all behavioral measures. Over the course of a block, progressively more false alarms occurred (*t*(27) = 1.74, *p* = 0.047), and RTs became longer (RT: *t*(27) = 2.47, *p* = 0.010; quintile: *t*(27) = 3.31, *p* = 0.001) and more variable (*t*(27) = 3.06, *p* = 0.003; Fig 1b). The proportion of misses also increased with time (*t*(27) = 3.31, *p* = 0.001), but misses were rare (0.2% of all trials) and will thus not be considered in any further analyses. In sum, over the course of a block performance deteriorated. For the sake of simplicity, we hereafter refer to the effect of time within blocks as ‘time-on-task’ effects.

### The effects of time-on-task on tonic pupil fluctuations

Having established that behavioral performance on the task degraded over time, we next turned to the pupil data. We applied a sliding window to the unsegmented pupil data that was identical to the one applied to the behavioral data (a width of 50 trials and a step size of 15 trials). We extracted two measures: 1) the average pupil diameter in each window, hereafter referred to as ‘baseline diameter’; and 2) the average temporal derivative of baseline diameter, which quantifies the extent to which the pupil tended to dilate or constrict within each window. The derivative measure was calculated as the average difference between each two consecutive samples within the window (using MATLAB’s ‘diff’ function). This is equivalent to the difference in baseline diameter between the first and last sample of the window. For each of the pupillary measures this resulted in a time series that was identical in length to the time series of the behavioral measures.

**Fig 3:**
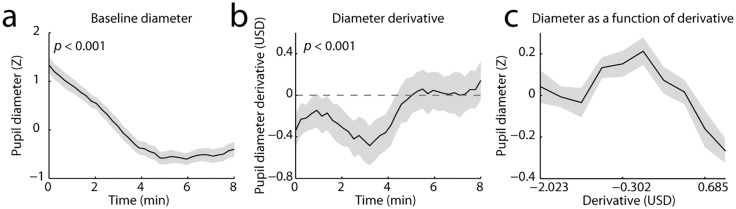
Baseline diameter and derivative. a) Time-on-task effect for baseline diameter, and b) for diameter derivative. *p-*values are listed in the top left corner of each panel. The derivative is shown as variance-normalized but without the mean removed. Values below the horizontal dotted line indicate that on average the pupil is constricting, whereas values above the line indicate that the pupil is dilating. c) The relationship between pupil diameter and its derivative. Baseline pupil diameter plotted as a function of the derivative. Diameter is smallest when the pupil is dilating the fastest. USD: Units standard deviation.

As a direct follow-up on the behavioral analyses, we first examined whether the pupillary measures also showed time-on-task effects. To do so, we fitted a straight line to each pupil time series. The slope of the fitted line was informative of linear trends over time. We averaged the slopes across blocks for each participant and compared the distribution of slopes to 0 using a two-tailed *t*-test. We had no clear hypothesis regarding the direction of the time-on-task effect for the derivative of pupil diameter, so for this test we also used a two-tailed *t*-test. Both pupil measures showed significant linear time-on-task effects. Over the course of a block baseline diameter became smaller (*t*(27) = 8.10, *p* < 0.001; Fig 3a). On average, its derivative was initially negative and became less negative over time (*t*(27) = 4.40, *p* < 0.001; Fig 3b), reflecting the fact that the pupil progressively decreased in diameter during the early-to-mid portions of a block and reached a relatively stable diameter thereafter.

Before examining the relationship between baseline diameter and its derivative vis-à-vis the behavioral performance measures, we wanted to make sure that the two pupil measures were not highly correlated with each other, so that they might be expected to explain unique variance in the behavioral measures. In order to clarify the relationship between baseline diameter and its derivative, we correlated their respective time series derived from the sliding-window approach, for each participant and each block, and compared the distribution of Fisher-transformed correlation coefficients averaged across blocks to zero using a *t*-test. Although the correlation was consistently negative across participants (*t*(27) = -2.48, *p* = 0.020; Fig 3c), the average correlation coefficient was rather small: -0.12. Thus, the two pupil measures only weakly co-varied (*R*^2^ < 1.5%) and their capacities to explain unique portions of the variance in behavior were high.

### The relationship between tonic pupil fluctuations and behavior

**Fig 4:**
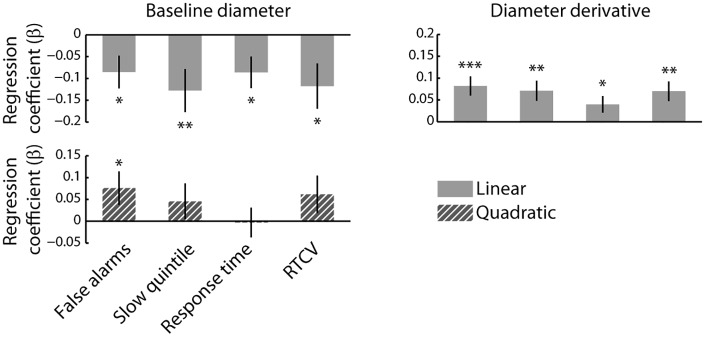
The relationship between pupil diameter and behavior. Regression coefficients are shown per pupil measure and behavioral measure. USD: Units standard deviation. Error bars represent the SEM. *: *p* < 0.05; **: *p* < 0.01; ***: *p* < 0.001.

We next used multiple regression to examine linear relationships between the time series of each of the Z-scored pupillary measures and each of the Z-scored behavioral measures, within participants and within blocks. A separate model was constructed for each of the pupillary/behavioral measure pairings. We also included quadratic regressors in these models, but only report the quadratic relationships between baseline diameter and the behavioral measures. The inclusion of quadratic regressors in the regression models for the derivative did not affect the direction and significance of the linear regression coefficients. We averaged the resulting regression coefficients across blocks for each participant and compared the distribution of regression coefficients to 0 using *t*-tests. We expected the typical Yerkes-Dodson relationship between baseline diameter and behavior (but see below), and therefore used one-tailed *t*-tests to compare the quadratic regression coefficients to zero. Furthermore, because all analyses concerning the derivative of baseline diameter were exploratory, we used two-tailed *t-*tests in these analyses. The linear and quadratic relationships between the pupil measures and the behavioral measures are summarized in Fig 4.

We found a significant positive quadratic relationship between baseline diameter and false alarm rate (*t*(27) = 1.99, *p* = 0.029), indicating that false alarm rate tended to increase at both the upper and lower extremes of baseline pupil diameter. This finding is consistent with the long-recognized inverted U-shaped relationship between arousal and task performance [22]. However, we found no such quadratic relationship for the other behavioral measures (all *p*s > 0.05).

**Fig 5:**
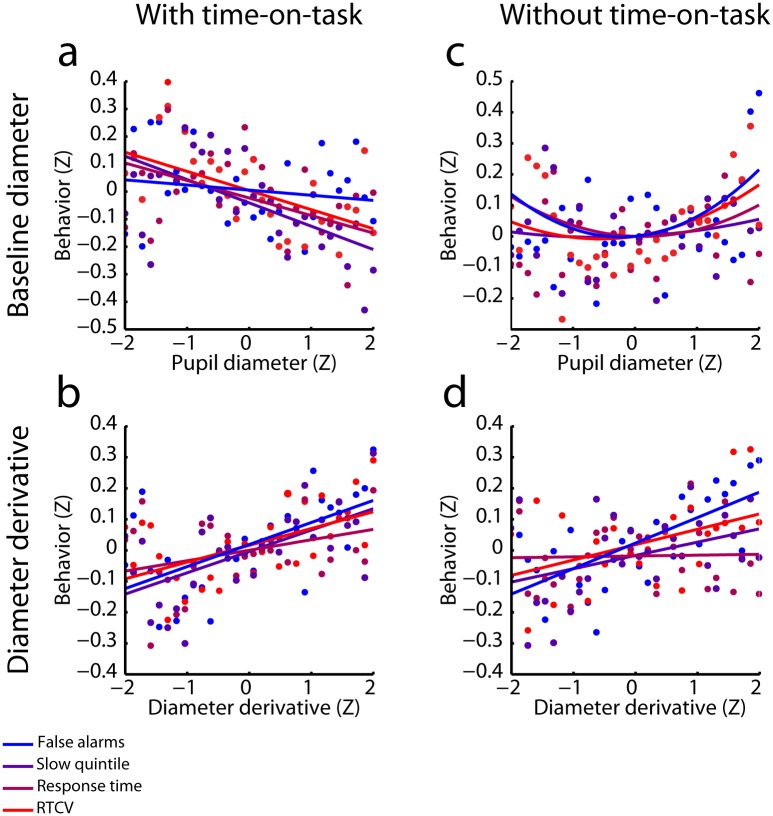
Relationship between pupillary measures and behavior, before (a,b) and after (c,d) regressing out time-on-task. Pupil data were z-scored within participants and blocks, aggregated across participants, and then divided up into 30 bins, and the behavioral data were sorted according to pupil diameter. Large positive values on the Y-axis indicate relatively poor behavioral performance. The initially linear relationship between baseline diameter and behavior becomes U-shaped after controlling for time-on-task, whereas the relationship between the derivative of baseline diameter and behavior remains linear after controlling for time-on-task. Straight lines are least squares regression lines, curved lines are fitted 2^nd^-order polynomials.

Given the linear time-on-task effects on baseline diameter and each of the behavioral measures, it may be expected that baseline diameter be linearly related to false alarm rate, RT, RTCV, and the proportion of trials that fell within the slowest RT quintile. We thus used one-tailed *t*-tests to test this hypothesis. In line with this notion, all behavioral measures were negatively related to baseline diameter (false alarm rate: *t*(27) = 2.28, *p* = 0.02; quintile: *t*(27) = -2.60, *p* = 0.008; RT: *t*(27) = -2.38, *p* = 0.012; RTCV *t*(27) = -2.27, *p* = 0.016). Thus, more false alarms and longer and more variable RTs tended to occur when baseline diameter was smallest which, as shown earlier, also tended to coincide with the end of task blocks. The linear relationships between baseline diameter and each of the behavioral measures are shown in Fig 5a.

Interestingly, the derivative of pupil diameter showed a significant positive linear relationship with all behavioral measures (false alarm rate: *t*(27) = 3.71, *p* = 0.001; quintile: *t*(27) = 3.10, *p* = 0.005; RT: *t*(27) = 2.10, *p* = 0.046; RTCV: *t*(27) = 3.11, *p* = 0.005). The positive relationship indicated that periods during which the pupil was relatively stable or dilating (i.e., the value of the derivative was positive/least negative) were characterized by the most false alarms and the slowest and most variable RTs (Fig 5b). In other words, periods in which the pupil showed little change in size over time or tended to dilate slowly, were marked by the poorest behavioral performance.

In order to rule out the possibility that these results were dependent on the choice of window size, we repeated the regression analysis for a range of sliding window sizes (40 s to 4 min, and an 8-s difference in width between each consecutive window size). For each window size, we then computed the regression coefficients indicating linear and quadratic relationships between the time series of each of the Z-scored pupillary measures and each of the Z-scored behavioral measures. We then averaged the resulting regression coefficients across blocks, and across the behavioral measures, and computed their area under the curve (AUC) across window sizes. This AUC summary statistic indicated whether on average the behavioral measures showed a relationship (linear or quadratic) with the two pupil measures. Finally, we tested if the group-level distribution of AUCs differed from 0 using one-tailed *t*-tests. If the linear pupil-behavior relationships were not dependent on the choice of a single (arbitrary) window size, we expected the AUC of the linear regression coefficients to go in the same direction as the initial regression coefficients. That is, we would expect the AUC to be negative for diameter, and positive for the derivative. As expected, the linear AUCs were significantly different from 0 and in the predicted direction for both pupil measures (diameter: *t*(27) = -2.62, *p* = 0.007; derivative: *t*(27) = 4.05, *p* < 0.001). Also in line with our expectations, the quadratic AUC for baseline diameter did not differ from zero (*t*(27) = 0.09, *p* = 0.47). Altogether, these results show that periods during which the pupil was smallest and remained relatively stable or dilated again were marked by the poorest behavioral performance on the task. These effects were consistent across a range of time scales.

### The relationship between tonic pupil fluctuations and behavior, controlled for time-on-task

It is possible that the relationships between baseline diameter and behavior reported above simply reflect the strong effects of time-on-task on these two types of variables, rather than a more intrinsic, time-invariant relationship. We therefore wondered whether shared effects of time-on-task on baseline diameter and behavior might be obscuring more subtle relationships between the associated measures. To address this possibility, we explored whether the relationship between the pupillary measures and behavior remained after statistically controlling for time-on-task. To do so, we performed similar regression analyses as before, except that we included a linearly increasing predictor that tracked time-on-task (i.e., the time elapsed within each block). As a result, the regression coefficients represented the relationship between the pupillary measures and behavior, independent of a linear time-on-task effect.

**Fig 6:**
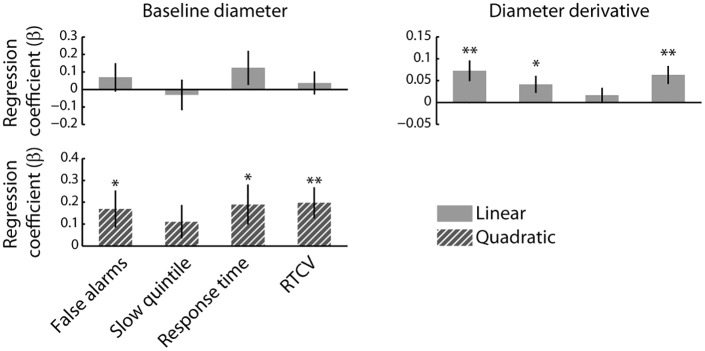
The relationship between pupil diameter and behavior, after statistically controlling for time-on-task. Regression coefficients per pupil measure and behavioral measure with time-on-task included as a variable of non-interest. Error bars represent the SEM. *: *p* < 0.05; **: *p* < 0.01.

As can be seen in Fig 6, the initial linear relationships between baseline diameter and the RT measures became quadratic when time-on-task was taken into account. Both relatively small and large diameters were associated with an increased false alarm rate, and slower and more variable RTs (false alarm rate: *t*(27) = 1.99, *p* = 0.028; quintile: *t*(27) = 1.45, *p* = 0.08; RT: *t*(27) = 2.06, *p* = 0.025; RTCV: *t*(27) = 2.79, *p* = 0.005), whereas linear relationships between pupil size and these behavioral measures were no longer present (all *p* > 0.2). This suggests that a U-shaped relationship between baseline diameter and RT measures was indeed initially obscured by strong time-on-task effects (Fig 5c). In contrast, the linear relationships between the derivative and the behavioral measures that were evident in the original regression models were largely preserved in the model that statistically controlled for time-on-task (false alarm rate: *t*(27) = 3.09, *p* = 0.005; quintile: *t*(27) = 2.13, *p* = 0.041; RT: *t*(27) = 0.93, *p* = 0.360; RTCV: *t*(27) = 3.07, *p* = 0.005; Fig 5d). These effects indicate that periods in which linearly detrended pupil diameter was generally increasing were associated with relatively impaired performance.

Again, these results were not dependent on the choice of window size, because the AUC summary statistics across window sizes and behavioral measures showed a similar shift from linear to quadratic for baseline diameter after statistically controlling for time-on-task (linear AUC: *t*(27) = 0.29, *p* = 0.39; quadratic AUC: *t*(27) = 3.08, *p* = 0.002). The linear relationship between the derivative and behavior was also preserved in the AUC across window sizes (linear AUC: *t*(27) = 3.08, *p* = 0.002).

Together, these results suggest that time-on-task was driving the initially observed linear relationships between mean baseline diameter and task performance, and to some extent obscured latent quadratic relationships between these variables. In contrast, the linear relationship between task performance and the derivative of pupil diameter was mostly robust to controlling for time-on-task. As we discuss below, this relationship likely reflects the quadratic relationship between diameter and behavior that occurred independent of time-on-task.

## Discussion

Using a fast-paced sustained attention task, we found robust linear relationships between baseline pupil diameter and several behavioral manifestations of attentional lapses. However, these linear relationships primarily reflected the joint effect of time-on-task on baseline pupil and behavior: as performance deteriorated over the course of a block (as indexed by increased false alarm rate and slower and more variable RTs), the pupil became progressively smaller. Importantly, when this effect of time-on-task was statistically partialled out, the relationship between baseline diameter and behavior became U-shaped: more false alarms, and longer and more variable RTs occurred during periods of both relatively small and relatively large baseline diameter, a pattern that is consistent with the Yerkes-Dodson law of mental task performance [22] and the adaptive gain theory of LC-NE function [3].

Previous studies on the relationship between pupil diameter and attentional state have yielded contradicting results. Some studies have reported that moments of poor task performance or off-task thought are associated with larger baseline diameter [6–9]. Conversely, others have reported that poor task performance is associated with smaller baseline diameter [10–14]. Our research suggests three methodological reasons for these mixed results. First, a multitude of measures have been used to assess attentional state. Although the performance measures used in the current study generally showed similar relationships with pupil diameter, there were some differences between the measures. For example, as opposed to the RT measures, false alarm rate already displayed a U-shaped relationship with diameter before the effect of time-on-task was partialled out. This discrepancy may be explained by the possibility that slow and variable RTs primarily reflect a decrease in attentional focus [34]—equivalent to a lower (e.g. [35]) and/or more variable [36] rate of decision formation—whereas false alarms may reflect either a decrease in attentional focus or an inadvertent lowering of the response threshold [37]. Thus these signatures of attentional lapses may have partially dissociable mechanistic bases. To make sure that key conclusions do not depend on the specific choice of measure, future studies should ideally use a range of performance measures, as we have done here. Second, the majority of previous studies have reported only categorical comparisons (e.g., on-task versus off-task thought [6,10,38]; or normal versus slow RTs [9,12]) to assess the relationship between pupil diameter and attentional state. However, such comparisons cannot reveal potential non-linear relationships between pupil and behavior. Thus, the manner in which the relationship between baseline diameter and attentional state is assessed restricts the conclusions that can be drawn from the data.

Finally, our results suggest that contradictory findings in the literature may also be due to differences between studies in the presence and nature of parallel effects of time-on-task on pupil diameter and behavior. In tasks that are demanding, such as our task, the dominant finding is that attentional lapses and mind wandering are associated with a smaller baseline pupil diameter than non-lapses or on-task thought (e.g. [11–13]). This pattern may simply be due to a progressive decrement in behavioral performance along with a monotonic decline in pupil diameter over time, perhaps reflecting a shift from center to left on the Yerkes-Dodson curve and a corresponding abandonment of exploitative behavior [3], or reduced top-down control of behavior [21]. Such time-dependent shifts on the Yerkes-Dodson curve could be the consequence of depleted cognitive resources. As noted by Hopstaken et al. [10], there is substantial overlap between the behavioral consequences of mental fatigue and the characteristics of low-arousal states. Nevertheless, the mechanistic origin of simultaneous effects of time-on-task on pupil diameter and performance remains an interesting open question for future research. In less demanding tasks, by contrast, time-related performance decrements are often less severe, and pupil diameter has even been reported to increase over time in such settings [15]. Such an absence of shared time-on-task effects might in turn afford greater scope for revealing more nuanced relationships between pupil diameter and task performance in the observed data. We suggest that future studies should carefully distinguish between pupil-behavior relationships due to time-on-task and potentially more subtle relationships that operate on a faster time scale. As we have shown, this dissociation can be easily achieved via the implementation of appropriate statistical control.

Aside from yielding insight into the mechanisms underlying attentional lapses, an important long-term goal of studies such as ours is to establish psychophysiological markers that can be used in on-line biofeedback systems, aimed at predicting and preventing lapses of attention. Recently, deBettencourt et al. [39] made an important step towards the realization of such a system. By providing participants with well-timed performance feedback based on the on-line analysis of brain imaging data, they could improve participants’ performance on a sustained attention task. However, the involvement of brain imaging equipment imposes obvious restrictions on the real-world applicability of this technique. Our results, however, indicate that the pupil could potentially be used to predict when lapses of attention are likely to occur. Given the relatively non-invasive and cost-effective nature of eye-tracking, such a system would offer substantial advantages over neuroimaging-based systems. However, it should be noted that the average regression coefficients that captured the relationship between the dynamics of the pupil and the dynamics of behavior, although consistent across participants, were modest in size (between 0.1 and 0.2). Thus, future work is needed to establish the practical feasibility of using pupil diameter and its derivative as on-line markers of attentional lapses.

Our findings that the average derivative of the pupil diameter time series was linearly related to behavioral performance, and that this relationship was independent of time-on-task, indicate that the derivative of pupil diameter offers a potential marker of attentional performance. The robustness of the derivative to time-on-task compared to baseline diameter may be explained by the way we computed this measure. Specifically, the derivative reflected the difference in baseline diameter between the first and last time point in the sliding window. Thus, this measure was less affected by block-wide trends in pupil diameter but instead captured changes at the temporal scale of the applied sliding window. Moreover, the derivative of a U-shaped signal is monotonically increasing (*f*(*x*) = a*x*^2^ + c → *f* ‘(*x*) = 2a*x*). The relationship between pupil diameter and its derivative are fixed in this way, because the latter is computed as a function of the former. Any quadratic relationship between a variable (e.g., baseline diameter) and another variable (e.g., behavior) will therefore be measurable as a linear relationship between the derivative of the first variable (baseline diameter derivative) and the second variable (behavior). This holds true even in the presence of a superimposed linear relationship between diameter and behavior (e.g., due to time-on-task effects), because the linear part of the function will simply reduce to a constant in the derivative (*f*(*x*) = a*x*^2^ + b*x* + c → *f* ‘(*x*) = 2a*x* + b). Thus, the linear relationship between the baseline diameter derivative and behavior likely reflected the quadratic relationship between baseline diameter and behavior that occurred independent of time-on-task.

A bio-feedback system could thus incorporate the derivative of pupil diameter and a receiver operating characteristic analysis could be performed to examine how reliably the signal preceding a behavioral response discriminates between lapse and non-lapse trials. Future studies could also incorporate purely momentary fluctuations in the derivative of the pupil (cf. [16,31]) as opposed to changes during a longer window. These instantaneous fluctuations are, however, beyond the scope of the current study, as we were primarily interested in tonic fluctuations that evolve over longer time periods and how they relate to global fluctuations in attentional performance. Such global fluctuations are more akin to real-world fluctuations in behavior in settings that require prolonged sustained attention, as when an air-traffic controller must monitor a display for long periods of time.

In conclusion, our results demonstrate that time-on-task, a factor that is often ignored in studies on the relationship between pupil diameter and attentional state, can obscure non-linear pupil-behavior relationships. The non-linear (inverted U-shaped) relationship between baseline pupil diameter and attentional performance that we observed after partialling out time-on-task effects is consistent with the adaptive gain theory of LC-NE function [3]. Finally, our results indicate that the derivative of pupil diameter is a potential marker of attentional performance that could be used for the on-line prediction and prevention of attentional lapses.
